# Function of Presynaptic Inhibitory Cannabinoid CB_1_ Receptors in Spontaneously Hypertensive Rats and Its Modification by Enhanced Endocannabinoid Tone

**DOI:** 10.3390/ijms25020858

**Published:** 2024-01-10

**Authors:** Marek Toczek, Eberhard Schlicker, Patryk Remiszewski, Barbara Malinowska

**Affiliations:** 1 Department of Experimental Physiology and Pathophysiology, Medical University of Białystok, Mickiewicza Str. 2A, 15-222 Białystok, Poland; patryk.remiszewski@umb.edu.pl (P.R.); barbara.malinowska@umb.edu.pl (B.M.); 2 Department of Pharmacology and Toxicology, University of Bonn, Venusberg-Campus 1, 53127 Bonn, Germany; e.schlicker@uni-bonn.de

**Keywords:** cannabinoid receptors, CP55940, endocannabinoids, pithed rat, presynaptic receptors, sympathetic system, spontaneously hypertensive rats

## Abstract

We studied whether the function of presynaptic inhibitory cannabinoid CB_1_ receptors on the sympathetic nerve fibres innervating resistance vessels is increased in spontaneously hypertensive rats (SHR) like in deoxycorticosterone (DOCA)–salt hypertension. An increase in diastolic blood pressure (DBP) was induced by electrical stimulation of the preganglionic sympathetic neurons or by phenylephrine injection in pithed SHR and normotensive Wistar–Kyoto rats (WKY). The electrically (but not the phenylephrine) induced increase in DBP was inhibited by the cannabinoid receptor agonist CP55940, similarly in both groups, and by the endocannabinoid reuptake inhibitor AM404 in SHR only. The effect of CP55940 was abolished/reduced by the CB_1_ receptor antagonist AM251 (in both groups) and in WKY by endocannabinoid degradation blockade, i.e., the monoacylglycerol lipase (MAGL) inhibitor MJN110 and the dual fatty acid amide hydrolase (FAAH)/MAGL inhibitor JZL195 but not the FAAH inhibitor URB597. MJN110 and JZL195 tended to enhance the effect of CP55940 in SHR. In conclusion, the function of presynaptic inhibitory CB_1_ receptors depends on the hypertension model. Although no differences occurred between SHR and WKY under basal experimental conditions, the CB_1_ receptor function was better preserved in SHR when the endocannabinoid tone was increased by the inhibition of MAGL or the endocannabinoid transporter.

## 1. Introduction

The pathogenesis of arterial hypertension, including primary hypertension which accounts for more than 90% of all hypertensive individuals, is complex and still requires intensive studies [1,2]. The sympathetic nervous system plays a fundamental role in the development and progression of hypertension [3,4]. The sympathetic overdrive augments plasma catecholamine levels which, in turn, increase vascular resistance and cardiac output [1]. An enhanced level of sympathetic nerve activity and increased intravascular fluid volume are particularly relevant for drug-refractory and drug-resistant hypertensive patients in whom hypertension is uncontrolled despite using ≥5 and ≥3 medications, respectively [2,5]. Therefore, novel adjunctive therapies targeting the sympathetic nervous system, such as renal denervation and carotid baroactivation, have been developed [3,4,5]. Nonetheless, the search for noninvasive pharmacological strategies modulating sympathetic outflow is justified as well.

Sympathetic tone is subject to the modulation of noradrenaline release from axon terminals by presynaptic receptors, which have been identified both in humans and animals [6,7,8,9]. Functional changes in the presynaptic receptors modulating noradrenaline release from (e.g., vascular) sympathetic nerve endings may participate in the development and/or maintenance of hypertension. Thus, an increased responsiveness of facilitatory presynaptic β-adrenergic [10,11], AT_1_ angiotensin [12] and angiotensin 1–7 receptors [13], and an impaired function of inhibitory presynaptic α_2_-adrenergic [11], A_1_ adenosine [14,15], endothelin-1 [16] and neuropeptide Y receptors [17], but enhancement of the function of inhibitory presynaptic γ-aminobutyric acid (GABA_B_) receptors [18] has been demonstrated in spontaneously hypertensive rats (SHRs), which are the most widely used animal model of primary hypertension [19,20].

Peripheral presynaptic cannabinoid CB_1_ receptors represent a potential target for antihypertensive therapy. They are located on pre- and/or postganglionic nerve fibres innervating resistance vessels and heart; their stimulation diminishes the electrically evoked increases in blood pressure (BP), heart rate (HR) and/or plasma noradrenaline levels in pithed rats [21,22,23] and rabbits [24,25] as well as rat and guinea-pig atria [26] and human atrial appendages [27].

Hypertension affects the endocannabinoid system, which consists of (1) cannabinoid receptors (e.g., CB_1_), (2) endogenous cannabinoids represented by anandamide (AEA) and 2-arachidonoylglycerol (2-AG) and (3) mechanisms terminating their activity including enzymes like fatty acid amide hydrolase (FAAH; degrades AEA) and monoacylglycerol lipase (MAGL; degrades 2-AG) and endocannabinoid membrane transporters (EMTs) [28]. Examples of endocannabinoid system overactivation in SHR (compared to Wistar–Kyoto rats (WKY), their normotensive controls include (1) higher levels of AEA and/or 2-AG in the blood plasma [29], aorta and small mesenteric arteries [30], (2) increased expression or coupling efficiency of CB_1_ receptors in the heart [31], aorta [30,31], small mesenteric arteries [30], brain [32], and (3) decreased EMT activity) [29]. In harmony with the latter findings, patients with diagnosed primary hypertension exhibited higher plasma concentrations of AEA and lower EMT activity [29].

Compounds enhancing endocannabinoid tone are suggested to possess a therapeutic potential in hypertension [28]. Indeed, acute intravenous administration of FAAH (URB597 and AM3506) and EMT (AM404 and OMDM-2) inhibitors reduced BP and/or HR in SHR but not in their normotensive controls [31,32]. However, chronic intraperitoneal URB597 administration for 2 weeks did not decrease BP in SHR [33]. By contrast, the dual FAAH/MAGL inhibitor JZL195, administered to SHR for 2 weeks revealed a weak hypotensive effect and prevented the progression of hypertension [34]. Thus, when comparing the outcomes of chronic treatment of SHR with URB597 (FAAH blockade only) and JZL195 (dual FAAH/MAGL blockade), increasing 2-AG levels by the inhibition of MAGL might become a new therapeutic option in hypertension.

In our previous study on pithed hypertensive deoxycorticosterone acetate (DOCA)–salt rats (an animal model of secondary hypertension), we have revealed an enhanced function of the inhibitory presynaptic cannabinoid CB_1_ receptors on the sympathetic nerves innervating the resistance vessels [35]. The aim of the present study was to examine how these receptors behave in pithed SHR. Additionally, using endocannabinoid degradation and re-uptake inhibitors, we investigated whether endogenously formed cannabinoids could modulate sympathetic tone via presynaptic CB_1_ receptors.

## 2. Results

### 2.1. General

Conscious SHR had a much higher systolic (SBP), mean (MBP) and diastolic (DBP) blood pressure as well as HR (179 ± 2, 160 ± 2, 151 ± 2 mmHg, and 372 ± 4 beats/min, respectively; *n* = 100; *p* < 0.001) than normotensive WKY rats (107 ± 2, 91 ± 2, 84 ± 2 mmHg, and 327 ± 4 beats/min, respectively; *n* = 106). In pithed and vagotomised rats treated with pancuronium (0.8 μmol/kg) and the vehicle for AM251 basal SBP, MBP, DBP and HR were also higher (*p* < 0.01) in SHR than in WKY. For DBP and HR values, see Table 1 (first line). SBP and MBP values in SHR (*n* = 37) were 79 ± 1 and 59 ± 1 mmHg, respectively, and in WKY (*n* = 36) 70 ± 1 and 51 ± 1 mmHg, respectively. The vehicle for JZL195 decreased HR in WKY rats, whereas the drugs under study had no influence on the basal cardiovascular parameters in either strain (Table 1).

**Figure 1:**
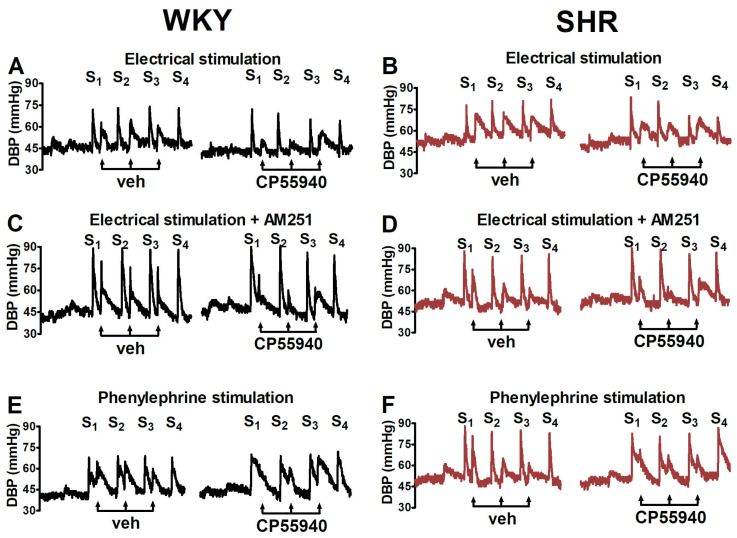
Original traces from representative experiments in which the influence of the cannabinoid receptor agonist CP55940 on the increase in diastolic blood pressure (DBP) induced by electrical stimulation (panels (**A**–**D**)) or by injection of phenylephrine (0.01 μmol/kg; panels (**E**,**F**)) in normotensive control Wistar–Kyoto rats (WKY) and spontaneously hypertensive rats (SHR) was studied. DBP was stimulated four times (S_1_–S_4_) at intervals of 7 min. For electrical stimulation of the preganglionic sympathetic nerve fibres, five pulses (0.75 Hz, 1 ms, 50 V) were administered. Increasing doses of CP55940 (0.01, 0.1 and 1 μmol/kg) or its vehicle (veh) were given 5 min before S_2_, S_3_ and S_4_. AM251 (3 μmol/kg; panels (**C**,**D**)) or its vehicle (panels (**A**,**B**,**E**,**F**)) was administered 10 min before S_1_. Each rat was injected with pancuronium (0.8 µmol/kg) 5 min before S_1_.

**Figure 2:**
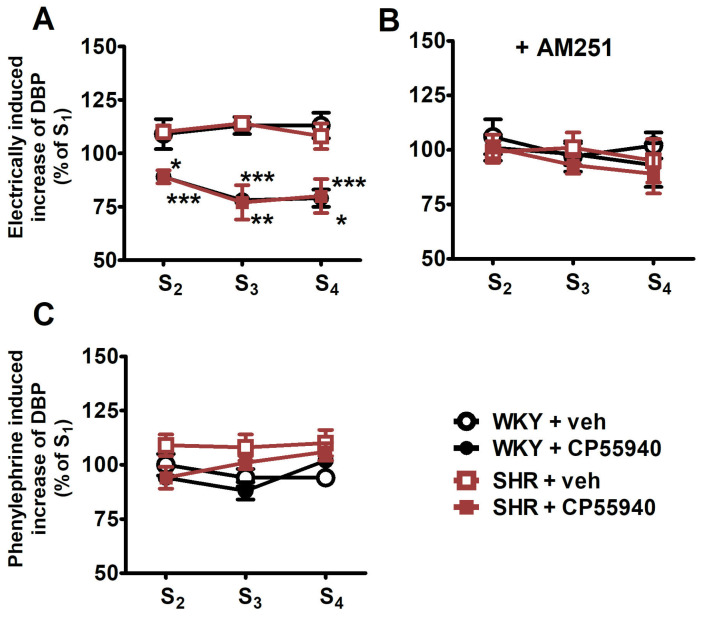
Panel (**A**): Influence of the cannabinoid receptor agonist CP55940 on the electrically (0.75 Hz, 1 ms, 50 V, 5 pulses) induced increase in diastolic blood pressure (DBP) in pithed and vagotomised spontaneously hypertensive rats (SHR) and normotensive Wistar–Kyoto rats (WKY). Panel (**B**): Effects of CP55940 in the presence of the CB_1_ receptor antagonist AM251. Panel (**C**): Effect of CP55940 on the phenylephrine (0.01 μmol/kg)-induced increase in DBP. Increasing doses of CP55940 (0.01, 0.1 and 1 μmol/kg) or its vehicle (veh) were given 5 min before S_2_, S_3_ and S_4_. AM251 (3 μmol/kg) or its vehicle was administered 10 min before S_1_. Each rat was injected with pancuronium (0.8 µmol/kg) 5 min before S_1_. Means ± SEM of n for panels (**A**–**C**), respectively: WKY + veh: *n* = 5, 5, and 6; for WKY + CP55940: *n* = 7, 8, and 7, for SHR + veh: *n* = 6, 5, and 7, and for SHR + CP55940: *n* = 7, 7, and 7; * *p* < 0.05, ** *p* < 0.01, *** *p* < 0.001 vs. respective group with vehicle for CP55940.

**Figure 3:**
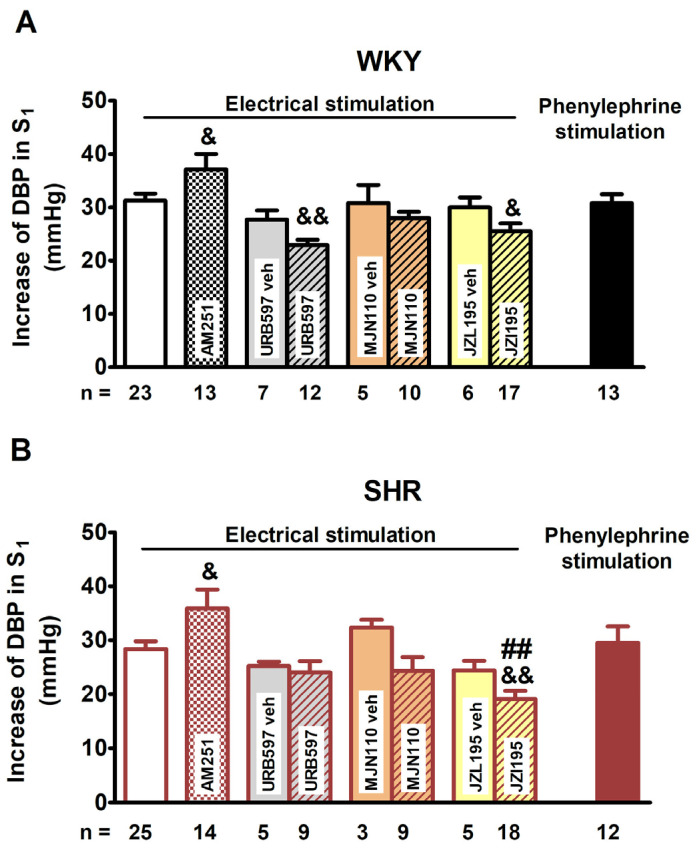
Increases in diastolic blood pressure (DBP) during the first stimulation (S_1_) induced electrically (0.75 Hz, 1 ms, 50 V, 5 pulses) or by phenylephrine (0.01 μmol/kg) in pithed and vagotomised normotensive Wistar–Kyoto rats (WKY; panel (**A**)) and spontaneously hypertensive rats (SHR; panel (**B**)). Each rat was injected with pancuronium (0.8 µmol/kg) 5 min before S_1_. Experiments were performed in the presence of the CB_1_ receptor antagonist AM251 (3 μmol/kg), the fatty acid amide hydrolase (FAAH) inhibitor URB597 (9 μmol/kg; i.p.), the monoacylglycerol lipase (MAGL) inhibitor MJN110 (3 μmol/kg; i.p.) and the dual FAAH/MAGL inhibitor JZL195 (46 μmol/kg; i.p.) or their respective vehicle (veh). Means ± SEM of *n* = 3–25; ^##^ *p* < 0.01 vs. the respective value in normotensive WKY group; ^&^ *p* < 0.05, ^&&^ *p* < 0.01 vs. the respective control group without antagonist or inhibitor or vehicle for inhibitor (white columns).

**Figure 4:**
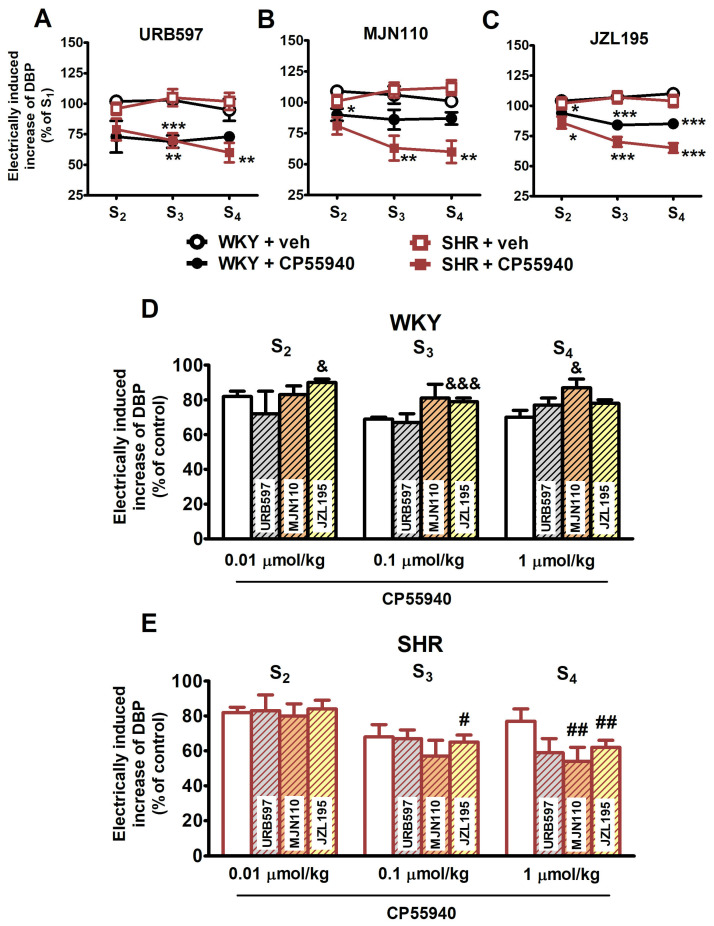
Influence of the fatty acid amide hydrolase (FAAH) inhibitor URB597 (9 μmol/kg, i.p.; panels (**A**,**D**,**E**)), the monoacylglycerol lipase (MAGL) inhibitor MJN110 (3 μmol/kg, i.p.; panels (**B**,**D**,**E**)) and the dual FAAH/MAGL inhibitor JZL195 (46 μmol/kg, i.p.; panels (**C**–**E**)) on the effect of CP55940 on the electrically induced increase in diastolic blood pressure (DBP) in pithed and vagotomised spontaneously hypertensive rats (SHR) and normotensive Wistar–Kyoto rats (WKY). Increasing doses of CP55940 (0.01, 0.1 and 1 μmol/kg) or its vehicle (veh) were given i.v. 5 min before S_2_, S_3_ and S_4_. Each rat was injected with pancuronium (0.8 µmol/kg; i.v.) 5 min before S_1_. Means ± SEM of n for panels (**A**–**C**), respectively: WKY + veh: *n* = 6, 5, and 10; for WKY + CP55940: *n* = 6, 5, and 7, for SHR + veh: *n* = 4, 5, and 10, and for SHR + CP55940: *n* = 5, 4, and 8; * *p* < 0.05, ** *p* < 0.01, *** *p* < 0.001 vs. respective group with vehicle for CP55940 (control); ^&^ *p* < 0.05, ^&&&^ *p* < 0.001 vs. respective group without inhibitors (white columns); ^#^ *p* < 0.05, ^##^ *p* < 0.01 vs. respective value in normotensive WKY group.

**Figure 5:**
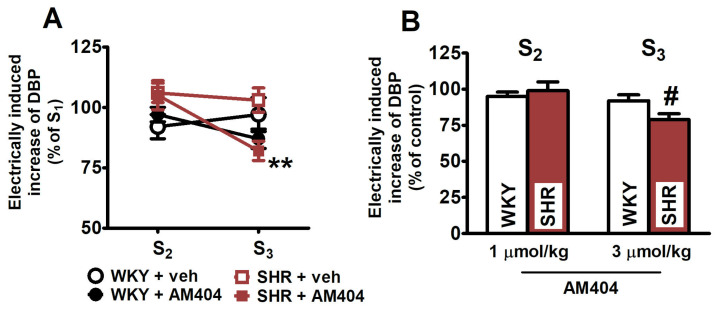
Influence of the endocannabinoid membrane transporter (EMT) inhibitor AM404 on the electrically (0.75 Hz, 1 ms, 50 V, 5 pulses) induced increase in diastolic blood pressure (DBP) in pithed and vagotomised spontaneously hypertensive rats (SHR) and normotensive Wistar–Kyoto rats (WKY). AM404 1 and 3 µmol/kg (i.v.) was given 5 min before S_2_ and S_3_, respectively; controls received vehicle instead. Each rat was injected with pancuronium (0.8 µmol/kg) 5 min before S_1_. The values are expressed as % of the first stimulation (S_1_; panel (**A**)) or as % of vehicle for AM404 control (panel (**B**)). Means ± SEM of *n* = 6 for WKY + veh, *n* = 5 for WKY + AM404, *n* = 6 for SHR + veh, and *n* = 6 for SHR + AM404; ** *p* < 0.01 vs. respective group with vehicle for AM404; ^#^ *p* < 0.05 vs. respective normotensive WKY group.

Electrical stimulation (0.75 Hz, 1 ms, 50 V, 5 pulses) of the preganglionic sympathetic nerve fibres innervating the resistance blood vessels induced comparable increases in DBP (S_1_) in WKY rats (31 ± 1 mmHg; *n* = 23) and in SHR (28 ± 1 mmHg, *n* = 25; Figure 1). Intravenous injection of phenylephrine (0.01 μmol/kg) increased DBP to the same level as electrical stimulation both in normo- and hypertensive animals (S_1_: 31 ± 2 mmHg, *n* = 13 and 30 ± 3, *n* = 12, respectively; Figure 1). The vasopressor responses both in hypertensive and normotensive animals not treated with the cannabinoid receptor agonist CP55940 or the EMT inhibitor AM404 did not markedly change upon repeated electrically or phenylephrine-evoked stimulations (S_2_–S_4_), which means the ratios S_2_/S_1_, S_3_/S_1_ and S_4_/S_1_ (expressed as a percentage) were close to 100–110% (Figure 2, Figure 3, Figure 4 and Figure 5). For original traces from representative experiments, see Figure 1.

### 2.2. Effects of the Cannabinoid Receptor Agonist CP55940 in the Presence and Absence of the CB_1_ Receptor Antagonist AM251

The cannabinoid receptor agonist CP55940 (0.01, 0.1 and 1 μmol/kg) caused a dose-dependent inhibition of the electrically induced increase in DBP in WKY rats—maximally by about 20% of S_1_ for its two highest doses (Figure 2A). Since the increases in DBP in the presence of vehicle for CP55940 (0.1 and 1 μmol/kg) were about 110% of S_1_, the maximal inhibitory effect of the cannabinoid receptor agonist expressed in % of control was about 30%. The inhibitory effect of CP55940 did not differ between WKY and SHR (Figure 2A; for original traces, see Figure 1A,B).

The cannabinoid CB_1_ receptor antagonist AM251 (3 μmol/kg) attenuated the inhibitory effect of CP55940 in WKY and SHR to about the same extent (Figure 2B; for original traces, see Figure 1C,D). AM251 (3 μmol/kg) enhanced the electrically induced increase in DBP (S_1_) by 20–25% in WKY and SHR (Figure 3; for original traces, see Figure 1C,D).

In contrast to the electrically evoked increases in DBP, CP55940 (0.01, 0.1 and 1 μmol/kg) failed to modify the rise in DBP induced by phenylephrine (0.01 µmol/kg) in WKY and SHR (Figure 2C; for original traces, see Figure 1E,F).

### 2.3. Effects of the Endocannabinoid Degradation Enzyme Inhibitors URB597, MJN110 and JZL195 and Their Influence on the CP55940 Action

The electrically induced increase in DBP (S_1_) was diminished by the FAAH inhibitor URB597 in WKY (by about 15% of the control group) and by the dual FAAH/MAGL inhibitor JZL195 in WKY and SHR (by about 15% and 20% of the respective control group, respectively). In addition, the MAGL inhibitor MJN110 tended to decrease S_1_ by about 10% and 25% in WKY and SHR, respectively. The vehicles for the enzyme inhibitors did not influence the electrically evoked increases in DBP in any group of rats (S_1_; Figure 3).

Inhibitors of endocannabinoid degradation affected the dose–response curve of CP55940 (0.01, 0.1 and 1 μmol/kg) for its inhibitory effect on the neurogenic vasopressor response in a different manner (Figure 4). Note that the results in Figure 4 are expressed in % of the respective S_1_ (Figure 4A–C) and in % of control (vehicle for CP55940; Figure 4D,E).

URB597 (9 µmol/kg) failed to modify the inhibitory influence of CP55940 on the neurogenic vasopressor response both in WKY and in SHR (Figure 4A,D,E). In contrast, MJN110 (3 µmol/kg) and JZL195 (46 µmol/kg) only tended to enhance the inhibitory effect of CP55940 (0.1 and 1 μmol/kg) in SHR (from about 70 to 55% of control; clearly visible in Figure 4B,C and, in comparison to the respective white columns, in Figure 4E) but attenuated its effect in WKY from about 70 to 80-85% of control (Figure 4; compare to the respective white columns in Figure 4D). The difference between the two strains (marked with # in Figure 4E) reached a significant level in the case of MJN110 (for dose of CP55940: 1 µmol/kg) and JZL195 (for doses of CP55940: 0.01 and 0.1 µmol/kg).

### 2.4. Effects of the Endocannabinoid Transporter Inhibitor AM404

The endocannabinoid transporter inhibitor AM404 did not alter the electrically induced increase in DBP in WKY and SHR at 1 μmol/kg (Figure 5A,B). However, its higher dose (3 μmol/kg) diminished the electrically evoked rise in DBP in SHR (by about 20%) without affecting this parameter in WKY (Figure 5A,B). Note that the effect of AM404 was examined in the absence of CP55940 only.

## 3. Discussion

The aim of the present study was to examine whether presynaptic vascular cannabinoid CB_1_ receptors on sympathetic nerve endings innervating resistance vessels are altered in spontaneously hypertensive rats (SHR), i.e., in a model of primary hypertension. In our previous study on DOCA–salt hypertensive rats, a model of secondary hypertension, the function of the presynaptic CB_1_ receptors was markedly increased [35]. Like in our study on DOCA–salt rats [35], we used pithed rats in which only peripheral neural mechanisms can be studied; note that cannabinoids have multiple effects on BP including those in the central nervous system, which might even cancel out peripheral inhibitory effects; for example, enhanced CB_1_ receptor-mediated neurotransmissions in the rostral ventrolateral medulla (RVLM) has been demonstrated to play a role in the development of obesity-induced hypertension in rats [36]. Animals received pentobarbitone for anaesthesia prior to pithing since this anaesthetic, unlike, e.g., urethane, does not interfere with presynaptic receptors [37]. Under the experimental conditions of our study, the electrically induced vasopressor response is predominantly related to the release of noradrenaline but does not involve an increase in cardiac output and catecholamine release from the adrenal medulla [21]. We have focused on DBP, which is dependent mainly on peripheral resistance, in order to allow for comparisons with our previous studies [21,35] in which changes in DBP were monitored as well.

In conscious SHR, blood pressure (SBP, MBP, DBP) was higher by 65–80% than in WKY, whereas HR was slightly increased only. These results resemble those in DOCA–salt rats in which DBP, compared to the corresponding control, the uninephrectomised (UNX) rat, was increased by about 65% and HR did not differ at all (Table 2; [35]). After pithing, DBP was slightly higher and HR was marginally higher in SHR than in WKY. An entire different picture emerged for DOCA–salt vs. UNX rats in which both DBP and HR were lower (!) by about 20% than in normotensive controls (Table 2). Shortly, as explained in our previous paper [35], the lower values of cardiovascular parameters in pithed and vagotomised DOCA–salt hypertension prove the crucial role of sympathetic tone in its pathogenesis, which is additionally enhanced by high salt intake. With respect to the vasopressor response induced by electrical stimulation and phenylephrine, no difference between SHR and WKY occurred. This is in very marked contrast to UNX and DOCA–salt rats in which the vasopressor response induced by electrical stimulation and by phenylephrine was lower by 60 and 20% (although the latter value was not statistically significant), respectively (Table 2; [35]). For identification of the presynaptic CB_1_ receptor, we used the same drugs as in our previous studies [21,35]. We applied the cannabinoid CB_1/2_ receptor agonist CP55940 [38] which inhibited the neurogenic vasopressor response in pithed rats [21,23,35] and rabbits [24] in a manner sensitive to CB_1_ receptor antagonists. In WKY animals, CP55940 led to a dose-dependent inhibition of the neurogenic vasopressor response (maximum inhibitory effect by 30%), and the effect was abolished by the CB_1_ receptor antagonist AM251. A comparable maximum degree of inhibition of the neurogenic vasopressor response was also obtained for CP55940 in UNX rats [35] and for CP55940 and WIN55,212-2 (another CB_1_/CB_2_ agonist) in normotensive pithed Wistar rats [21]. The fact that CP55940 did not affect the vasopressor response elicited by the α_1_-adrenoceptor agonist phenylephrine shows that the CB_1_ receptors are indeed located presynaptically on the pre- or postganglionic sympathetic nerve fibres.

The dose–response curves of CP55940 for its inhibitory effect on the neurogenic vasopressor response coincided in SHR and WKY; this held true both in the absence and presence of AM251. By contrast, the maximum CP55940-induced inhibition was by 50% in the DOCA–salt rats and by 30% in the UNX control rats ([35]; Table 2). Since the low basal level of the electrically neurogenic vasopressor response might have influenced the CP55940-induced inhibition in DOCA–salt rats, additional experimental series in which a high basal level of the neurogenic vasopressor response occurred were considered in the study by Toczek et al. [35]. However, the marked difference in the effect of CP55940 was retained, and one has to conclude that CB_1_ receptor function is very differently affected by hypertension in both experimental models.

Given alone, AM251 increased the neurogenic vasopressor in SHR and WKY by about 20–25%. The reason for the facilitatory effect may be that AM251 interrupts the inhibitory effect of endogenously formed cannabinoids accumulating in the biophase of the presynaptic CB_1_ receptors. Another explanation might be that the presynaptic CB_1_ receptors are constitutively active, i.e., they inhibit noradrenaline release even in the absence of an agonist, and that AM251, which is an inverse CB_1_ receptor agonist, counteracts the constitutive activity and eventually leads to a facilitation of transmitter release [39,40]. In marked contrast to the present study, AM251 facilitated the neurogenic vasopressor response in hypertensive DOCA–salt rats without affecting it in normotensive UNX animals [35]. The exclusive facilitatory effect in the hypertensive strain agrees well with numerous studies in which the activity of the endogenous cannabinoid system is increased under hypertension (for a review of the literature, see the Section 1).

The present study shows that presynaptic CB_1_ receptors do not contribute to the development/maintenance of hypertension in SHR. One might have expected such a role for SHR since, as mentioned in detail in the Section 1, some facilitatory presynaptic receptors showed increased activity, and some inhibitory receptors showed decreased activity in this model of hypertension. In the DOCA–salt model, the function of the presynaptic CB_1_ receptor was even increased, and this alteration suggests that the CB_1_ receptor does not facilitate but rather counteracts the pathophysiological events leading to/maintaining hypertension.

**Table 2:** Cardiovascular parameters in primary (SHR) and secondary (DOCA–salt) hypertension in comparison to the respective normotensive animals.

Parameter/Experimental Condition	Change in SHR vs. WKY	Ref. to SHR	Change in DOCA-Salt vs. UNX	Ref. to DOCA
Cardiovascular parameters in pithed rats (if not stated otherwise)		present paper		[35]
BP (conscious)	↑		↑	
BP	↑		↓	
HR (conscious)	↑		↔	
HR	↑		↓	
Electrically induced increase in DBP (S_1_)	↔		↓	
Phenylephrine induced increase in DBP (S_1_)	↔		↓	
Function of presynaptic inhibitory CB_1_ receptors (inhibitory effect of cannabinoid agonist CP55940)	↔		↑	
Influence of FAAH and/or MAGL or endocannabinoid reuptake inhibitors on BP in conscious rats				
URB597 or AM3506 (acute)	↓	[31,32]	ND	
URB597 (chronic)	↔	[33]	↔ ^a^/↓ ^b^	
JZL195 (acute)	↓	[34]	ND	[41]
JZL195 (chronic)	↓	[34]	ND	
AM404 or OMDM-2 (acute)	↓	[31]	ND	
AEA level				
plasma	↓ ^c^ ↑ ^d^	^c^ [42], ^d^ [29]	↑	[42]
aorta/mesenteric G3	↑	[30]	↔/ND	[30]
heart	↓	[42]	↑	[42]
2-AG level				
plasma	↓	[42]	↑	[42]
aorta/mesenteric G3	↑	[30]	↔/ND	[30]
heart	↓	[42]	↑	[42]
CB_1_ receptor density				
aorta/mesenteric G3	↑	[30]	↑	[30]

^a^ 4 week old, ^b^ 6–7-week-old rats, both at the beginning of the hypertension induction procedure; ^c^ 8–10-week-old, ^d^ 16-week-old rats. ↑ increase; ↓, decrease; ↔, no effect. 2-AG, 2-arachidonoylglycerol; AEA, anandamide; AM404, endocannabinoid reuptake inhibitor; AM3506, FAAH inhibitor; BP, blood pressure; DBP, diastolic blood pressure; DOCA–salt, deoxycorticosterone acetate–salt rats; FAAH, fatty acid amide hydrolase; HR, heart rate; JZL195, dual FAAH/MAGL inhibitor; MAGL, monoacylglycerol lipase; mesenteric G3, segments of mesenteric arteries with a diameter of 250 µm; ND, not determined; OMDM-2, endocannabinoid reuptake inhibitor; S_1_, first stimulation; SHR, spontaneously hypertensive rats; UNX, uninephrectomised rats; URB597, FAAH inhibitor; WKY, Wistar–Kyoto rats.

As listed in Table 2, the enhanced function of the presynaptic inhibitory CB_1_ receptors on the sympathetic nerve endings innervating resistance vessels in DOCA–salt as opposed to SHR is not the only difference in the endocannabinoid system between these models of primary and secondary hypertension. Thus, plasma and cardiac levels of AEA and 2-AG, the two major endocannabinoids, were lower in SHR but higher in DOCA–salt in comparison to the respective normotensive controls [42]. Moreover, the aorta levels of AEA and 2-AG were increased in SHR but not changed in DOCA–salt [30]. However, Li et al. [29] found a higher plasma concentration of AEA in SHR, but this group used older rats than Biernacki et al. [42] (16-week-old and 8–10-week-old animals, respectively); in the current study, we used 10–12-week-old SHR. The above difference points to age-dependent changes within the same hypertension model. Consequently, the chronic administration of URB597 diminished BP and HR in older but not in younger DOCA–salt rats (i.e., 6–7 and 4 weeks old at the beginning of the hypertension induction procedure, [41]) and failed to modify both cardiovascular parameters in SHR [33].

Since the endogenous cannabinoid tone is mainly increased in hypertension (see above), it was tempting to study the impact of a further increase in the activity of this system on CB_1_ receptor function in SHR. Note that AEA and 2-AG are degraded by the enzymes FAAH and MAGL, respectively; both endocannabinoids are removed from the biophase also by an endocannabinoid transporter [28]. The FAAH inhibitor URB597, which after acute intravenous administration lowered BP in anaesthetized SHR [31], failed to influence the dose–response curve of CP55940 in SHR or WKY; the same result had been obtained for DOCA–salt and UNX rats [35]. Another three drugs targeting the endocannabinoid system (which had not been examined in our previous study [35]) affected the CB_1_ receptor-mediated effect in SHR and WKY in a very different manner. AM404, an inhibitor of the endocannabinoid transporter, inhibited the neurogenic vasopressor response in SHR but only tended to do so in WKY, whereas MJN110, a MAGL inhibitor, and JZL195, a combined MAGL/FAAH inhibitor, attenuated the effect of CP55940 in WKY but tended to enhance it in SHR.

The attenuation of the dose–response curve of CP55940 in WKY may be explained as follows. If the concentration of endocannabinoids is increased in the biophase of the CB_1_ receptor, the subsequent activation of this receptor by an exogenously added agonist will be blunted. A similar situation exists for the noradrenergic system [43]. Thus, the effect of an α_2_-adrenoceptor agonist, e.g., clonidine, will be attenuated if the endogenous concentration of noradrenaline is simultaneously increased by inhibition of the neuronal noradrenaline transporter. The reason that the CB_1_ receptor function was better preserved in SHR than in WKY may be that the vascular expression of CB_1_ receptors [30] and/or the efficiency of CB_1_ receptor coupling [32] are higher and the plasma AEA and 2-AG levels are lower [42] in SHR than in WKY.

The present findings with MJN110, JZL195 and URB597 are in harmony with previous results in which enzyme inhibitors were administered chronically. JZL195 reduced BP in SHR but not in WKY [34], whereas URB597 failed to diminish BP in either strain ([33]; Table 2). These findings are compatible with the view that 2-AG probably plays a more important role in the regulation of the cardiovascular system in hypertension than AEA. Importantly, 2-AG levels in the resistance mesenteric G3 artery and aorta are 6 and 120 times higher than the respective values of AEA, respectively [30].

## 4. Materials and Methods

### 4.1. Animals

All surgical procedures and experimental protocols were performed in accordance with the European Directive (2010/63/EU) and Polish legislation and were approved by the local Animal Ethics Committee in Białystok and Olsztyn (Poland). Rats were obtained from the Center for Experimental Medicine of the Medical University of Białystok (Poland). Experiments were performed on male 10–12-week-old SHR and appropriate normotensive control WKY rats. Animals had free access to tap water and food pellets and were maintained under a 12/12 h light/dark cycle. The total number of animals in the experiments was 100 for SHR and 106 for WKY.

### 4.2. Conscious Rats

Systolic and mean blood pressure (SBP and MBP) and HR were measured in restrained conscious rats by a noninvasive tail-cuff method using a Rat Tail Blood Pressure Monitor (Hugo Sachs Elektronik-Harvard Apparatus, March-Hugstetten, Germany). Diastolic blood pressure (DBP) was calculated from the equation DBP = (3 × MBP − SBP)/2.

### 4.3. Pithed Rats

Procedures and laboratory equipment were similar as in our previous studies, e.g., [35]. Briefly, rats were anaesthetised intraperitoneally (i.p.) with pentobarbitone sodium (300 µmol/kg or ~70 mg/kg) and then injected with atropine (2 µmol/kg or ~1 mg/kg; i.p.). Following anaesthesia, animals were tracheotomised for artificial ventilation (10 mL/kg; 60 strokes/min) by using a respiratory system (7025 rodent ventilator; Ugo Basile, Comerio, Italy). Then, rats were bilaterally vagotomised and pithed by inserting a stainless-steel rod through the orbit and foramen magnum into the vertebral canal. The pithing rod was enamelled except for a uncovered segment situated at vertebrae T_2_-L_6_. In most experiments, the pithing rod was used as an electrode for electrical stimulation of the preganglionic sympathetic nerve fibres, leaving the spinal canal to study the electrically evoked increases in BP (known as neurogenic vasopressor response). For stimulation, an electrical field was generated between the pithing rod in the vertebral column and an indifferent electrode placed under the skin by means of a stimulator (Stimulator V type STV100, Bio-Sys-Tech, Białystok, Poland). DBP and SBP were measured from the right carotid artery via a transducer (ISOTEC; Hugo Sachs Elektronik-Harvard Apparatus GmbH). MBP was calculated from the equation MBP = (2 × DBP + SBP)/3. Heart rate was recorded from the ECG by means of subcutaneous electrodes. Body temperature was maintained constant at approximately 36 to 37 °C by using a heating pad (Bio-Sys-Tech, Białystok, Poland). Most drugs were administered intravenously (i.v.) through a femoral vein with the exception of endocannabinoid degradation inhibitors, which were administered intraperitoneally. After 15–25 min of equilibration, during which the cardiovascular parameters were allowed to stabilize, experiments were performed.

### 4.4. Experimental Protocol

**Figure 6:**
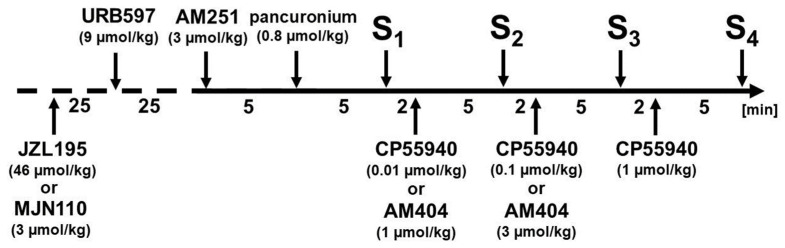
Experimental protocol used to examine the function of presynaptic CB_1_ receptors in pithed and vagotomised spontaneously hypertensive rats (SHR) and their normotensive control Wistar–Kyoto rats (WKY). Increases in diastolic blood pressure (DBP) were evoked four times (S_1_–S_4_) at intervals of 7 min by electrical stimulation (0.75 Hz, 1 ms, 50 V, 5 pulses) of the preganglionic sympathetic nerve fibres or by injection of the α_1_-adrenoreceptor agonist phenylephrine (0.01 μmol/kg; i.v.). Each animal received pancuronium (0.8 µmol/kg; i.v.) 5 min before S_1_. Increasing doses of the cannabinoid receptor agonist CP55940 (0.01, 0.1 and 1 µmol/kg; i.v.) or the endocannabinoid transport inhibitor AM404 (1 and 3 µmol/kg; i.v.) or their vehicles were given 5 min before subsequent stimulations (except for S_1_). The cannabinoid CB_1_ receptor antagonist AM251 (3 μmol/kg; i.v.) or its vehicle was injected 10 min before S_1_. The fatty acid amide hydrolase (FAAH) inhibitor URB597 (9 μmol/kg; i.p.), the monoacylglycerol lipase (MAGL) inhibitor MJN110 (3 μmol/kg; i.p.) and the dual FAAH/MAGL inhibitor JZL195 (46 μmol/kg; i.p.) or their respective vehicles were given 35, 60 and 60 min before S_1_, respectively. The numbers on the axis refer to the time intervals (in minutes) elapsing between two subsequent procedures (marked by arrows).

The experimental protocol is presented in Figure 6. Increases in BP were induced four times (S_1_–S_4_) by electrical stimulation (0.75 Hz, 1 ms, 50 V, 5 pulses) of the preganglionic sympathetic nerve fibres innervating the resistance blood vessels or by administration of the α_1_-adrenoreceptor agonist phenylephrine (0.01 µmol/kg or ~2 µg/kg; i.v.). Ten minutes before, S_1_ rats were injected with the CB_1_ receptor antagonist AM251 (3 µmol/kg or ~1.7 mg/kg; i.v.) or its vehicle. Five minutes later, animals were treated with pancuronium (0.8 µmol or ~0.5 mg/kg; i.v.) to avoid twitches associated with electrical stimulation. It was also administered to rats stimulated with phenylephrine to ensure identical experimental conditions. Stimulations S_2_-S_4_ were administered at intervals of 7 min. Increasing doses of the cannabinoid receptor agonist CP55940 (0.01, 0.1 and 1 µmol/kg or ~4, 40 and 400 µg/kg, respectively; i.v.) or its vehicle were given 5 min before S_2_, S_3_ and S_4_, respectively. Drug doses were like in our previous studies, e.g., [35]. In another series of experiments, the endocannabinoid transport inhibitor AM404 (1 and 3 µmol/kg or ~0.4 and 1.2 mg/kg, respectively; i.v.) or its vehicle were given 5 min before S_2_ and S_3_, respectively. We were not able to perform a fourth stimulation due to a sustained decrease in basal DBP following the i.v. injection of AM404 at a dose of 10 µmol/kg (like in the study of Iring et al. [44]). The FAAH inhibitor URB597 (9 µmol/kg or ~3 mg/kg), the MAGL inhibitor MJN110 (3 µmol/kg or ~1.4 mg/kg) and the dual FAAH/MAGL inhibitor JZL195 (46 µmol/kg or ~20 mg/kg) or their solvents were given i.p. 35, 60 and 60 min before S_1_, respectively. The dosage regimens for URB597 [45], MJN110 [46], and JZL195 [47] were chosen according to studies in which the same or similar doses were found to be effective.

### 4.5. Statistical Analysis

Statistical analysis was performed using Graph Pad Prism 5 (GraphPad Software Inc., La Jolla, CA, USA). Results are expressed as means ± SEM (*n* = number of animals). In order to quantify the effects of the agonist on the stimulated increases in DBP, the ratios S_2_/S_1_, S_3_/S_1_ and S_4_/S_1_ were determined. For better visualisation of the inhibitory effect, these ratios were expressed as percentages of the corresponding ratios obtained from the vehicle-treated animals (% of control). Student’s *t*-test for unpaired data was used to compare the mean values. When two or more treatment groups were compared to the same control, a one-way analysis of variance (ANOVA) followed by the Dunnett test was used. Differences were considered significant when *p* < 0.05.

### 4.6. Materials

Drugs and chemicals were obtained from the following sources: atropine sulphate, Cremophor EL, dimethyl sulfoxide (DMSO), 5-(1,1-dimethylheptyl)-2-[5-hydroxy-2-(3-hydroxypropyl)-cyclohexyl]-phenol (CP55940), 2-hydroxypropyl-β-cyclodextrin (cyclodextrin), 2,5-dioxopyrrolidin-1-yl 4-(bis(4-chlorophenyl)methyl)piperazine-1-carboxylate (MJN110), pancuronium bromide, (R)-(-)-phenylephrine hydrochloride and Tween-80 from Sigma-Aldrich (Steinheim, Germany); N-(piperidin-1-yl)-5-(4-iodophenyl)-1-(2,4-dichlorophenyl)-4-methyl-1H-pyrazole-3-carboxamide (AM251), N-(4-hydroxyphenyl)-5Z,8Z,11Z,14Z-eicosatetraenamide (AM404) and Tocrisolve 100 from Tocris Cookson Inc. (Bristol, UK); (3′-(aminocarbonyl)[1,1′-biphenyl]-3-yl)-cyclohexylcarbamate (URB597) from Cayman Chemical Company (Ann Arbor, MI, USA); 4-[(3-phenoxyphenyl)methyl]-1-piperazinecarboxylic acid 4-nitrophenyl ester (JZL195) from MedChemExpress (Monmouth Junction, NJ, USA); ethanol from POCH (Gliwice, Poland); pentobarbitone sodium from Biowet (Puławy, Poland); and sodium chloride (NaCl) from Chempur (Piekary Śląskie, Poland). Drugs were dissolved in saline with the following exceptions: AM251 in a mixture of DMSO, ethanol and Cremophor EL (2:1:1) and then diluted in saline (1:9) immediately before the experiment; CP55940 in a 19% *w*/*v* solution of cyclodextrin in saline immediately before the experiment; URB597 in a mixture of DMSO and Tween 80 (1:2) and then diluted in saline (3:7) immediately before the experiment; MJN110 in a mixture of ethanol and Tween 80 (1:2) and then diluted in saline (1:9) immediately before the experiment; JZL195 in a mixture of ethanol and Tween 80 (3:1) and then diluted in saline (1:8) immediately before the experiment. Drugs were administered in a volume of 2 mL/kg (pentobarbitone sodium and atropine), 1 mL/kg (inhibitors of endocannabinoid degradation) and 0.5 mL/kg (other drugs).

## 5. Conclusions

The function of inhibitory CB_1_ receptors located on the sympathetic nerve fibres innervating resistance vessels of pithed and vagotomised rats is dependent on the model of hypertension. As the present study suggests, it does not differ between SHR and WKY. A similar result was obtained for the inhibitory histamine H_3_ receptor [48]. These findings are somewhat surprising, since the function of other types of inhibitory presynaptic receptors was lower and that of facilitatory presynaptic receptors was higher in SHR than in WKY (for references, see the Section 1). All findings are in marked contrast to our previous study [35] in which the maximum extent of CB_1_ receptor-mediated inhibition was increased by two-thirds in the DOCA–salt when compared to the UNX rat, suggesting that the CB_1_ receptor does not support but rather impairs the development and/or maintenance of hypertension. Although no differences occurred between SHR and WKY under basal experimental conditions, CB_1_ receptor function was better preserved in SHR when the endocannabinoid tone was increased by the inhibition of MAGL or the transport of endocannabinoids.

## Figures and Tables

**Table 1:** Basal diastolic blood pressure (DBP) and heart rate (HR) in pithed and vagotomised spontaneously hypertensive rats (SHR) and normotensive Wistar–Kyoto rats (WKY).

	WKY			SHR		
Group	*n*	DBP (mmHg)	HR (beats/min)	*n*	DBP (mmHg)	HR (beats/min)
	36	43 ± 1	283 ± 4	37	49 ± 1 ^###^	294 ± 3 ^#^
+AM251	13	39 ± 1	275 ± 6	14	52 ± 1 ^###^	291 ± 6
+vehicle for URB597	7	49 ± 2	275 ± 4	5	50 ± 1	282 ± 10
+URB597	12	47 ± 1	270 ± 6	9	51 ± 1 ^##^	299 ± 10 ^#^
+vehicle for MJN110	5	41 ± 2	292 ± 4	3	49 ± 1 ^#^	296 ± 14
+MJN110	10	43 ± 1	279 ± 6	9	49 ± 2 ^###^	303 ± 8 ^#^
+vehicle for JZL195	6	45 ± 4	256 ± 9 ^&^	5	52 ± 2	317 ± 11 ^##^
+JZL195	17	40 ± 2	266 ± 5 ^&^	18	50 ± 1 ^###^	296 ± 6 ^###^

Basal diastolic blood pressure (DBP) and heart rate (HR) were measured before the first stimulation (S_1_). Each rat was injected with pancuronium (0.8 µmol/kg) 5 min before S_1_. Experiments were performed in the presence of the CB_1_ receptor antagonist AM251 (3 μmol/kg), the fatty acid amide hydrolase (FAAH) inhibitor URB597 (9 μmol/kg; i.p.), the monoacylglycerol lipase (MAGL) inhibitor MJN110 (3 μmol/kg; i.p.) and the dual FAAH/MAGL inhibitor JZL195 (46 μmol/kg; i.p.) or their respective vehicles. Means ± SEM; ^#^ *p* < 0.05, ^##^ *p* < 0.01, ^###^ *p* < 0.001 vs. respective normotensive group (WKY); ^&^ *p* < 0.05 vs. the respective value in the first line.

## Data Availability

The data presented in this study are available on request from the corresponding author. The data are not publicly available due to privacy.
